# Diagnosis of post-transplant coronary artery disease using contrast-enhanced coronary vessel wall imaging at 3.0 Tesla

**DOI:** 10.1186/1532-429X-14-S1-P138

**Published:** 2012-02-01

**Authors:** Tarique Hussain, Matthew Fenton, Sarah A Peel, Andrea Wiethoff, Rene M Botnar, Michael Burch, Gerald F Greil

**Affiliations:** 1 Division of Imaging Sciences, King's College London, London, UK; 2 Paediatric Cardiology, Great Ormond Street Hospital, London, UK; 3 Philips Healthcare (UK), Guildford, UK

## Summary

Coronary plaque characterization at 3.0 Tesla holds great potential for clinical benefit. Coronary allograft vasculopathy (CAV) occurs after heart transplantation and is an immune-mediated diffuse coronary intimal disease. We demonstrate the use of contrast-enhanced inversion-recovery prepared coronary vessel wall MRI at 3T for diagnosis. We further validate the technique in 23 patients with intravascular ultrasound (IVUS), showing an accuracy of 91%. Direct non-invasive imaging has great potential benefit for screening and prevention in these patients. The study has further significant implications for conventional coronary atherosclerosis.

## Background

Coronary Allograft Vasculopathy (CAV) remains the leading cause of late death after heart transplantation in children but is poorly detected by X-ray angiography. Intravascular ultrasound (IVUS) is invasive and costly, which precludes close follow-up. We have previously demonstrated the feasibility of MRI late gadolinium enhancement (LGE) in the coronary vessel wall to detect and grade CAV. The purpose of this study was to demonstrate the clinical utility of the technique and if it could be improved by imaging at 3.0 Tesla.

## Methods

Participants underwent IVUS and cardiac MRI. Maximal intimal thickness (MIT), mean intimal index (MII = mean intimal area/ mean vessel area) and Stanford grade (0 to 4(severe)) were recorded on IVUS. MRI included a coronary magnetic resonance angiogram (CMRA) of the left coronary artery and LGE vessel wall imaging after administration of 0.2 mmol/kg Gadolinium. Participants were randomised to 3T or 1.5T (Achieva, Philips Healthcare, Best, Netherlands). 32-element cardiac phased-array receiver coils were used. The mean enhancement diameter (ED) and index (Ei= mean enhancement area/ mean vessel area on MRI) was quantified on LGE images fused with CMRA as a roadmap. Additionally, images were scored for inter/intra-observer error in analysis and image quality (0 (not seen) to 4 (sharp image)). Location of LGE on MRI and location of significant intimal thickening on IVUS were also recorded for comparison.

## Results

24 adolescents participated in the study (characteristics: Table 1). One MRI failed due to patient discomfort. Overall, there was excellent correlation of MRI with IVUS. Pearson’s correlation for ED with MIT was 0.80 (p<0.001) and for Ei with MII was 0.92 (p<0.001). Correlation coefficients at 3T were similar to 1.5T (0.77 & 0.96 versus 0.81 & 0.62 respectively (all p<0.05)). Bland-Altman analysis also shows similar inter/intra-observer agreement between the two field strengths.

**Table 1:** Patient Characteristics

	1.5 TESLA	3.0 TESLA
Number of Patients	12	11
Male (n=)	5	5
Age (years)	15.1 ± 2.2 years	15.8 ± 1.7 years
Weight (kg)	58.9 ± 20.9	57.7 ± 10.9
Height (cm)	162 ± 9	164 ± 11
HR (bpm)	81 ± 8	88 ± 8
MIT (mm)	0.50 ± 0.29	0.98 ± 0.56
MII (%)	17.5 ± 5.7	24.7 ± 13.5
ED (mm)	5.38 ± 2.39	7.86 ± 6.57
Ei	0.53 ± 0.42	1.72 ± 2.35
Stanford Grade 4 disease (n=)	2	5
Previous CMV infection (n=)	2	1
Hypertension (n=)	10	7
Donor Age (years)	23.3 ± 11.9	25.1 ± 16.1

Where appropriate, mean ± standard deviation is given.

**Figure 1:**
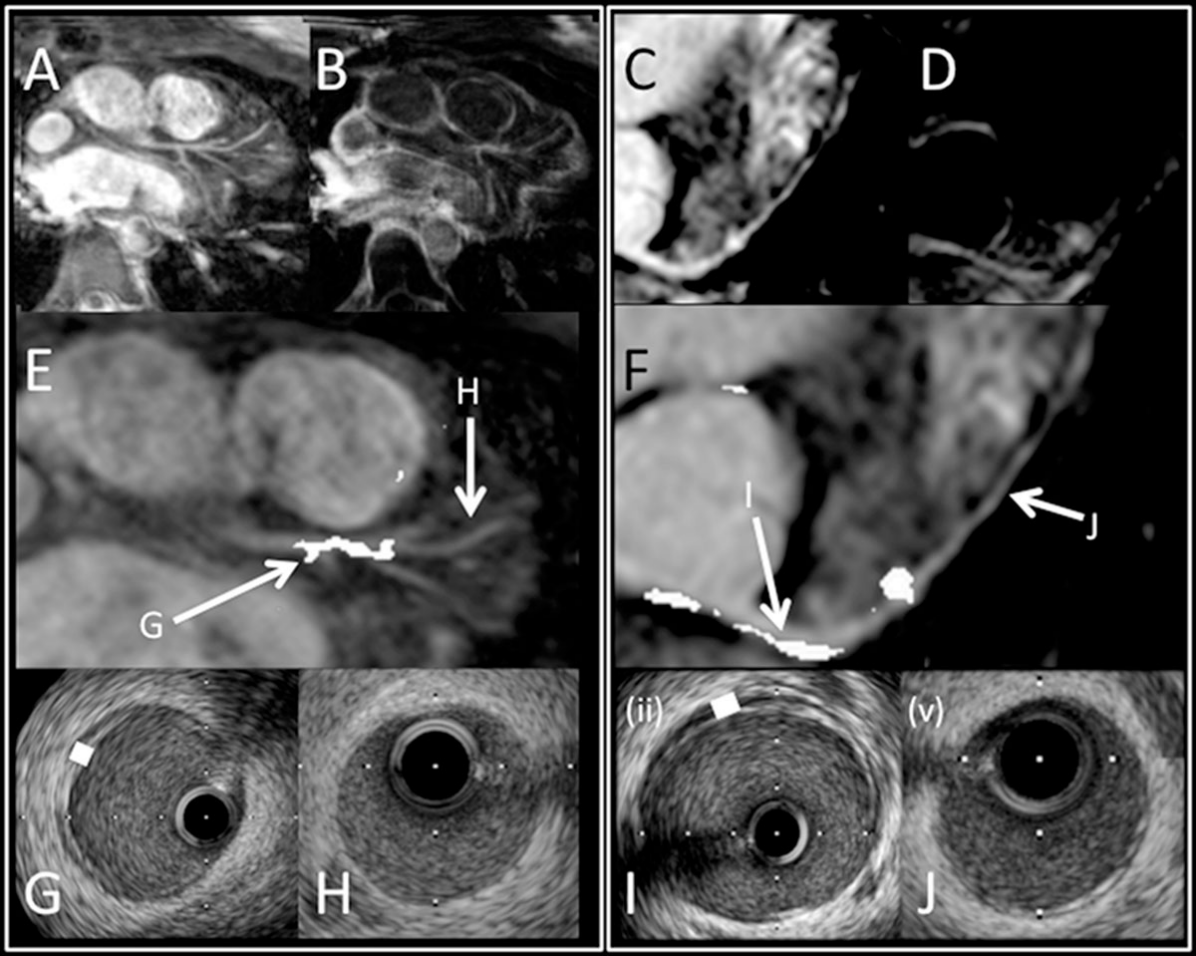
A) Patient 1 Left Coronary system CMRA at 3T. B) Patient 1 LGE at 3T. C) Patient 2 Left Coronary CMRA at 1.5T. D) Patient 2 LGE at 1.5T E) Overlay of Patient 1 LGE on CMRA at 3T. Arrows show corresponding positions for IVUS pictures G & H. F) Overlay of Patient 2 LGE on CMRA at 1.5T. Arrows show corresponding positions for IVUS pictures I & J. G & I) IVUS images. White box illustrates intimal thickening corresponding to enhancement on overlay picture above J & H) IVUS images. No significant intimal thickening corresponding to areas without enhancement on overlay picture above

However, LGE image quality was improved at 3T (median=3) compared to 1.5T (median=2; p=0.019 by Mann-Whitney test). Furthermore, at 1.5T only 11 out of 17 areas of enhancement on MRI were associated with corresponding significant disease on IVUS (i.e. location match to within 1mm from a major branch-point). At 3T, however, 11 of 13 enhancing lesions had exact and anatomical match (Figure 1).

Overall, the receiver operating characteristics curve demonstrates that a cut-off of 7.5 mm ED on MRI has 86% sensitivity and 94% specificity for the detection of significant (Stanford grade 4 on IVUS) CAV. This gives a positive predictive value of 86%, negative predictive value of 94% and accuracy of 91%.

In the multivariate analysis, donor age, length of time post transplant and MRI (ED) were the only significant independent predictors of maximum intimal thickness on IVUS.

## Conclusions

Coronary vessel wall delayed enhancement MRI is a valuable and accurate non-invasive method to quantify CAV. Improved image quality at 3.0 Tesla further ameliorates the accuracy of this technique.

## Funding

Funding: We would like to thank the Well Child Foundation (Cheltenham, UK) for financial support of the Senior Clinical Lecturer position of Gerald F. Greil, MD. The authors acknowledge financial support from the Department of Health via the National Institute for Health Research (NIHR) comprehensive Biomedical Research Centre award to Guy’s & St Thomas’ NHS Foundation Trust in partnership with King’s College London and King’s College Hospital NHS Foundation Trust. The MRI scanner is partly supported by Philips Healthcare. A. Wiethoff is an employee of Philips Healthcare, Best. All the other authors were not consultants or employees for Philips Healthcare and had control of inclusion of any data and information that might present a conflict of interest for A. Wiethoff.

No financial support was provided for data collection and analysis or manuscript preparation.

